# A case series of adenosine deaminase 2 deficient patients emphasizing treatment and genotype-phenotype correlations

**DOI:** 10.1186/1546-0096-13-S1-P62

**Published:** 2015-09-28

**Authors:** ED Batu, O Karadag, EZ Taskiran, U Kalyoncu, I Aksentijevich, M Alikasifoglu, S Ozen

**Affiliations:** 1 Hacettepe University Faculty of Medicine, Department of Pediatrics, Division of Rheumatology, Ankara, Turkey; 2 Hacettepe University Faculty of Medicine, Department of Internal Medicine, Division of Rheumatology, Ankara, Turkey; 3 Hacettepe University Faculty of Medicine, Department of Medical Genetics, Ankara, Turkey; 4 National Institutes of Health, National Human Genome Research Institute, Inflammatory Disease Section, Bethesda, USA

## Introduction

Deficiency of adenosine deaminase 2 (DADA2) causes a vasculopathy with autoinflammatory features associated with mutations in *CECR1*. The phenotype of DADA2 varies from only cutaneous lesions to full-blown systemic disease with central nervous system (CNS) involvement and aneurysms in visceral arteries which may overlap with the spectrum of polyarteritis nodosa (PAN).

## Objective

Our aim was to assess the characteristics of our patients with DADA2.

## Patients and methods

This is a descriptive case series of Turkish patients diagnosed with DADA2 at Hacettepe University. We performed Sanger sequencing in order to sequence 10 exons of *CECR1*.

## Results

We report six DADA2 patients with homozygous p.G47R mutation in *CECR1*. All were initially diagnosed as PAN (one cutaneous, others systemic) fulfilling the classification criteria for the disease and all but one having necrotizing arteritis lesions at skin biopsy and two had arterial aneurysms. All patients had a childhood onset of disease (median age 7.2 years). All had skin lesions varying from livedo racemosa to necrotic ulcers on fingers. There were recurrent fever and abdominal pain attacks in our patients. Four had CNS involvement; three in the form of strokes and one had borderline intelligence. Two patients had strabismus and one had optic neuritis. Two of the patients were sibs and these patients had low IgM. There was autoantibody positivity in two patients. Two patients had hematological involvement, one in the form of macrophage activation syndrome and one myelofibrosis. One of our patients had focal segmental glomerulosclerosis while another patient had renal AA type amyloidosis. All patients were refractory to corticosteroid treatment. One patient with extensive systemic amyloidosis was resistant to immunosuppressive and plasma treatments and died due to necrotizing pneumonia. One had prolonged remission on colchicine. Two responded to etanercept (one partially), one to mycophenolate mofetil (the patient with a previous diagnosis of cutaneous PAN), and one plasma treatment (temporarily). Literature review revealed that patients with homozygous p.G47R mutation have fewer strokes and predominantly PAN-like phenotypes compared to the patients with other mutations.

## Conclusion

DADA2 may be classified as a secondary vasculitis due to probable cause. Genotype-phenotype correlation may exist in DADA2 and etanercept may be a promising treatment; however, longer follow-up and prospective studies are needed.

